# Clinical Presentation of *T.b. rhodesiense* Sleeping Sickness in Second Stage Patients from Tanzania and Uganda

**DOI:** 10.1371/journal.pntd.0000968

**Published:** 2011-03-01

**Authors:** Irene Kuepfer, Emma Peter Hhary, Mpairwe Allan, Andrew Edielu, Christian Burri, Johannes A. Blum

**Affiliations:** 1 Swiss Tropical and Public Health Institute, Basel, Switzerland; 2 University of Basel, Basel, Switzerland; 3 Kaliua Health Centre, Kaliua, Tanzania; 4 Lwala Hospital, Lwala, Uganda; George Washington University, United States of America

## Abstract

**Background:**

A wide spectrum of disease severity has been described for Human African Trypanosomiasis (HAT) due to *Trypanosoma brucei rhodesiense (T.b. rhodesiense)*, ranging from chronic disease patterns in southern countries of East Africa to an increase in virulence towards the north. However, only limited data on the clinical presentation of *T.b. rhodesiense* HAT is available. From 2006-2009 we conducted the first clinical trial program (Impamel III) in *T.b. rhodesiense* endemic areas of Tanzania and Uganda in accordance with international standards (ICH-GCP). The primary and secondary outcome measures were safety and efficacy of an abridged melarsoprol schedule for treatment of second stage disease. Based on diagnostic findings and clinical examinations at baseline we describe the clinical presentation of *T.b. rhodesiense* HAT in second stage patients from two distinct geographical settings in East Africa.

**Methodology/Principal Findings:**

138 second stage patients from Tanzania and Uganda were enrolled. Blood samples were collected for diagnosis and molecular identification of the infective trypanosomes, and *T.b. rhodesiense* infection was confirmed in all trial subjects. Significant differences in diagnostic parameters and clinical signs and symptoms were observed: the median white blood cell (WBC) count in the cerebrospinal fluid (CSF) was significantly higher in Tanzania (134cells/mm^3^) than in Uganda (20cells/mm^3^; p<0.0001). Unspecific signs of infection were more commonly seen in Uganda, whereas neurological signs and symptoms specific for HAT dominated the clinical presentation of the disease in Tanzania. Co-infections with malaria and HIV did not influence the clinical presentation nor treatment outcomes in the Tanzanian study population.

**Conclusions/Significance:**

We describe a different clinical presentation of second stage *T.b. rhodesiense* HAT in two distinct geographical settings in East Africa. In the ongoing absence of sensitive diagnostic tools and safe drugs to diagnose and treat second stage *T.b. rhodesiense* HAT an early identification of the disease is essential. A detailed understanding of the clinical presentation of *T.b. rhodesiense* HAT among health personnel and affected communities is vital, and awareness of regional characteristics, as well as implications of co-infections, can support decision making and differential diagnosis.

## Author Summary

Sleeping sickness, or Human African Trypanosomiasis (HAT), caused by *Trypanosoma brucei rhodesiense* is one of the most neglected tropical diseases. It affects mainly rural, poor East African populations and has very high socio-economic impacts. *T.b. rhodesiense* HAT is an acute disease; patients quickly progress from the first stage, where trypanosomes are detectable in blood and lymph, to the second stage, where parasites penetrate the central nervous system. If left untreated, *T.b. rhodesiense* HAT is fatal. Disease control is hampered by the absence of sensitive diagnostic tools and safe drugs. Second stage patients can only be treated with melarsoprol, a highly toxic, arsenical drug. It is more difficult to treat patients successfully at advanced stages of the disease, and late onset of treatment should be avoided. Yet, most patients are treated for other conditions prior to HAT diagnosis. Therefore, it is important that health personnel in *T.b. rhodesiense* endemic regions have a detailed understanding of the clinical presentation of the disease and consider regional characteristics of *T.b. rhodesiense* HAT for decision making and differential diagnosis.

## Introduction

Human African Trypanosomiasis (HAT), also known as sleeping sickness, is caused by the protozoan parasites *T.b. gambiense* (West and Central Africa) and *T.b. rhodesiense* (East and South Africa). The disease is transmitted by tsetse flies (*Glossina ssp*.) predominantly in the rural areas of most of sub Saharan Africa. 60 Million people live at risk of infection, but less than 10% are under adequate surveillance [1], reflecting its neglected status. Sleeping sickness caused by either subspecies presents in two disease stages defined as the first, or haemo-lymphatic stage and the second, meningo-encephalitic stage. Diagnosis of HAT is made in blood, lymph and the cerebrospinal fluid (CSF). The second stage of the disease is indicated by the presence of trypanosomes and/or an elevated white blood cell (WBC) count (≥5WBC/mm^3^) in the CSF. The disease stage and the causative species of infection direct the choice of treatment. *T.b. gambiense* infections are treated with pentamidine in the first stage and eflornithine, a combination of eflornithine and nifurtimox, or melarsoprol in the second stage [2]–[4]. *T.b. rhodesiense* first and second stage infections are treated with suramin and melarsoprol respectively [2]. In the field, the trypanosome subspecies is entirely determined by the geographical location of the patient as the distinction of *T.b. gambiense* and *T.b. rhodesiense* is only possible in well equipped laboratories through PCR analysis. The detection of the human serum resistance-associated (SRA) gene unequivocally identifies *T.b. rhodesiense* trypanosomes [5], [6]. In Uganda, the only country where both forms of the disease are present, a potential geographical overlap of the two endemic areas has become likely [7]. This would hamper determination of infective trypanosomes under field conditions and therefore also the identification of the correct treatment.

For first stage infections there are no specific clinical signs and symptoms in both forms of the disease; fever, headache and loss of appetite are common. In *T.b. rhodesiense* the presence of a chancre at the site of the infective bite may be indicative for a trypanosome infection [8]. Second stage infections show disease-characteristic neuro-psychiatric signs and symptoms: severe endocrinological and mental disturbances and severe motor problems are the main signs [9]. While often considered together, Gambiense and Rhodesiense HAT are clinically and epidemiologically different diseases [10]. *T.b. gambiense* HAT is a chronic disease, whereas *T.b. rhodesiense* is characterized by an acute disease progression. If left untreated, both forms of HAT are fatal. The mean time to reach the second stage has been estimated at over one year for *T.b. gambiense* [11] but only 3 weeks for *T.b. rhodesiense* HAT [12]. Correspondingly, average times from infection to death are almost 3 years and 6 to 12 months, respectively [11], [12].

A diversity of forms of clinical progression from asymptomatic to acute have been reported for *T.b. gambiense* infections [13]–[15]. This seems to be even more pronounced for *T.b. rhodesiense* infections; a wide spectrum of disease severity ranging from a chronic disease pattern in southern countries of East Africa with existing reports of asymptomatic carriers [16] to an increase in virulence towards the north had been described [17]. Even though those differences were already described more than 60 years ago [18] the first comparative study was carried out in 2004: on the basis of the SRA gene polymorphism, trypanosomes isolates from Uganda (acute profile) and Malawi (chronic profile) confirmed to be of different genotypes. However, clinical characteristics of the study groups were limited the presence of a chancre and the self-reported duration of illness [19]. Another hypothesis postulates that the differences in disease severity could be attributed to differences in genetic resistance to trypanosomiasis among host populations [18].

From the estimated 50′000 to 70′00 cases per year [20], over 97% are *T.b. gambiense* cases and only a few thousand are due to *T.b. rhodesiense* [1]. Therefore, most literature concentrates on *T.b. gambiense* HAT. Its clinical picture and related cardiac and endocrinological disorders have been extensively described [21]–[28]. On the other hand, literature on the clinical aspects of *T.b. rhodesiense* HAT is scarce. We identified four studies (see table 1) describing its clinical presentation. Only one study in 60 patients infected with *T.b. rhodesiense* was designed prospectively and used a standardized questionnaire [29].

**Table 1:** Published literature on clinical signs & symptoms of *T.b. rhodesiense* HAT.

	Buyst/1977 [18]	Boatin/1986 [29]	Wellde/1989 [35]	Mbulamberi/1987 [36]
Number of patients	385	60	96	3152
Country	Zambia	Zambia	Zambia	Uganda
Disease stage of patients	2nd stage	2nd stage	2nd stage	1st stage^a^
Male/female ratio	N.A.	1.73	1.53	1.1
Chancre	N.A.	5	15.6	19
Headache	66.2	73.3	95.8	95.8
Fever	31.2	71.7	36.4	96.8
Lymphadenopathy	80.5	N.A.	86.4	17.6
Itching or pruritus	N.A.	35	53.1	N.A.
Oedema of face	30.1^b^	21.7	3.1	27.5
Swelling of legs	N.A.	43.3	25.3	N.A.
Joint pains	N.A.	65	88.5	95
Day time sleep	N.A.	63.3	70.8	26.8^c^
Night time sleep	N.A.	28.3	N.A.	N.A.
Abnormal coordination	N.A.	N.A.	51	N.A.
Abnormal speech	N.A.	N.A.	38.5	N.A.
Mental confusion	17.4	N.A.	N.A.	N.A.

N.A: not applicable;

a 98.7% of the patients were in the first stage and 1.3% of the patients in the second stage of the disease;

b reported as oedema,

c reported as somnolence

In this paper we describe the clinical presentation of second stage *T.b. rhodesiense* HAT in 138 patients from two distinct geographical settings in East Africa. We compare our findings to the existing literature and discuss factors that could explain the differences observed.

## Materials and Methods

### Study sites

The Kaliua Health Centre (KHC), a 50-bed missionary hospital in Tanzania (Urambo District) and the Lwala Hospital, a designated 100- bed district hospital in Uganda (Kaberamaido District) participated in the Impamel III program (improved application of melarsoprol).

### Study design and data collection

A proof-of-concept trial (n = 60) followed by a utilization study (n = 78) to assess the safety and efficacy of the abridged, 10-day melarsoprol schedule for the treatment of second stage HAT [30], [31] in *T.b. rhodesiense* patients.

Eligible for enrolment were second stage patients with a minimum age of 6 years and confirmed second stage HAT. Patients with first stage infections, pregnant women and moribund or unconscious patients were excluded. Patients were passively enrolled at the study sites.

Diagnosis of HAT was made in blood and in CSF. Blood was examined using microscopy and/or the haematocrit centrifugation technique [32]. If trypanosomes were present, a lumbar puncture was performed for disease staging. Analysis of the CSF was done by direct microscopy and/or single modified centrifugation technique and white blood cell (WBC) count using counting chambers. Second stage infections were confirmed by the presence of trypanosomes and/or ≥5 WBC/mm^3^ in the CSF. The standard assessment of co-infections included malaria, filariasis and voluntary testing for HIV/AIDS.

The local Principal Investigators filled individual case report forms (CRFs). Data used for describing the clinical presentation of the disease were patient demographics, diagnostic findings, self reported duration of illness and clinical signs and symptoms on admission graded by scale of severity (grade 0, 1, 2).

### Ethics statement

Each participant gave written informed consent. For the participation of children and adolescents (below 18 years) the parents, the legal representative or the guardian gave written informed consent. Ethical clearances were obtained from the Ethics Committees in Tanzania (National Institute for Medical Research), Uganda (Ministry of Health) and Switzerland (Ethics Committee of both cantons of Basel). Before first patient enrolment, the Impamel III program was registered in the database of Current Controlled Trials (ISRCTN40537886).

### Data management and statistical analysis

All data were double entered and verified using Epi Data 3.1 software (www.epidata.dk) and analysis was accomplished with the statistical software package STATA Version IC10.0 (STATA, StataCorp, USA). The statistical analysis was performed comparing proportions with the Pearson Chi Square and means with the Student's *t* test. Logistic regression was used to test differences between groups of patients with different co-infections.

## Results

The use of the abridged 10-day melarsoprol schedule for the treatment of second stage *T.b. rhodesiense* HAT was highly satisfactory (detailed safety and efficacy data to be published separately). In this paper we describe the clinical presentation of the disease in 138 second stage patients from Tanzania and Uganda. The majority of patients were passively detected. Nine (9) patients from Uganda (13%) were actively identified during a survey of the National Agricultural Research Organisation (NARO) in the HAT endemic region of the country. There was no significant difference between actively and passively recruited patients for the median WBC count in the CSF (actively detected: median WBC = 27, IQR = 24; passively detected: median WBC = 19, IQR = 43, p = 0.067) and the median self-reported duration of illness (actively detected: median = 3 months, IQR = 2, passively detected: median = 2 months, IQR = 4, p = 0.141). 14 patients (11 in Uganda and 3 in Tanzania) could not be examined per protocol as they died or were in a comatose state upon arrival at the study sites which led to an exclusion of those patients from the Impamel III trials.

By molecular analysis of blood samples, the presence of the SRA gene [33] was demonstrated and confirmed *T.b. rhodesiense* infection in all trial subjects [34].

Data on the demographic and diagnostic baseline characteristics of the study population are shown in table 2. The proportion of male (57.2%) and female (42.8%) patients was comparable. 18.8% (26/138) trial participants were younger than 16 years whereof 88.5% (23/26) were enrolled in Uganda. There were no county-specific differences for the presence of trypanosomes in blood and CSF: 99% (68/69) of patients from Tanzania and 91% (63/69) from Uganda had trypanosomes in blood (p = 0.0524) and 70% (55/69) and 86% (59/69) respectively had trypanosomes in the CSF (p = 0.3690). However, there was a significant difference for the median WBC count in the CSF in Tanzania and Uganda (134 vs. 20 WBC/mm^3^, p<0.0001). Also, a body mass index (BMI) below 16.5 was more frequent in patients from Uganda (p<0.0001).

**Table 2:** Demographic and diagnostic baseline characteristics.

	Total (n = 138)		Tanzania (n = 69)		Uganda (n = 69)	
	n	%	n	%	n	%
Age (years), mean ± SD	35±19		38±15		32±22	
Age (years), range (min.-max.)	6–85		9–70		6–85	
Male female ratio	1.34		1.38		1.3	
Age below 16 years	26	14	3	4	23	33
BMI^a^ (kg/m^2^) - mean ± SD	18.5±3.4		19.6±2.5		17.3±3.8	
BMI<16.5	38	28	5	7	33	48
Malaria positive on admission	57	41	55	80	2	3
Trypanosomes in blood	131	95	68	99	63	91
Trypanosomes in CSF^b^	114	83	55	80	59	86
WBC^c^ count in CSF						
Median	70		134		20	
Mean ± SD	86±82		135±85		37±40	
0–20 cells/ul - no. (%)	35	25	0		35	51
21–100 cells/ul - no. (%)	52	38	23	33	29	42
>100 cells/ul - no. (%)	51	37	46	67	5	7
Patients excluded^d^	14	N.A	3	N.A	11	N.A
Death upon arrival	6	N.A	3	N.A	3	N.A
Comatose upon arrival	8	N.A	0	N.A	8	N.A

N.A: not applicable;

a Body Mass Index,

b CSF: cerebrospinal fluid,

c WBC: white blood cell,

d exclusions due to other reasons (first stage infection, pregnancy) not shown

Clinical signs and symptoms reported at baseline and the level of significance (95%) are summarized in table 3. Headache, fever, general body pain and joint pains were common in both study populations. Clinical suspicion for cardiac insufficiency was found in both countries: 5.1% (7/138) of the patients had indication for left heart insufficiency (combination of cough and dyspnoe) and 5.8% (8/138) for right heart insufficiency (combination of oedema and hepatomegaly). Patients in Uganda had a more unspecific presentation of the disease whereas specific signs and symptoms for second stage HAT, namely sleeping disorders and aggressiveness were more common in patients from Tanzania.

**Table 3:** Clinical signs and symptoms at baseline and treatment outcomes.

	Total (n = 138)		Tanzania (n = 69)		Uganda (n = 69)		p-value
	n	%	n	%	n	%	
*Clinical signs & symptoms*							
Lymphadenopathy	27	19.6	7	10.1	20	29.0	0.0053
General body pain	132	95.7	69	100.0	63	91.3	0.0123
Headache	128	92.8	65	94.2	63	91.3	0.5114
Fever (≥37.5°C)	37	26.8	20	29.0	17	24.6	0.5643
Fever (>38.5°C)	4	2.9	0	0.0	4	5.8	0.0424
Joint pains	129	93.5	67	97.1	62	89.9	0.0847
Diarrhea	9	6.5	1	1.4	8	11.6	0.0158
Pruritus	21	15.2	4	5.8	17	24.6	0.0003
Oedema	40	29.0	26	37.7	14	20.3	0.0244
Dyspnoe	10	7.2	1	1.4	9	13.0	0.0086
Cough	27	19.6	8	11.6	19	27.5	0.0183
Tremor	54	39.1	43	62.3	11	15.9	0.0001
Hepatomegaly	25	18.1	4	5.8	21	30.4	0.0002
Splenomegaly	51	37.0	11	15.9	40	58.0	0.0001
Walking difficulties	75	54.3	35	50.7	40	58.0	0.3928
Abnormal movements	36	26.1	31	44.9	5	7.2	<0.0001
Sleeping disorder daytime	105	76.1	66	95.7	39	56.5	<0.0001
Sleeping disorder night time	88	63.8	64	92.8	24	34.8	<0.0001
Strange behaviour	25	18.1	15	21.7	10	14.5	0.2691
Disturbed appetite	120	87.0	60	87.0	60	87.0	1
Inactivity	100	72.5	57	82.6	43	62.3	0.0076
Speech impairment	16	11.6	6	8.7	10	14.5	0.2875
Aggressiveness	45	32.6	43	62.3	2	2.9	<0.0001
*Treatment outcomes*							
Death	15	10.1	7	10.1	8	11.6	0.7845
Cure (parasitological & clinical)	123	89.1	62	89.9	61	88.4	0.7845

**Figure 1:**
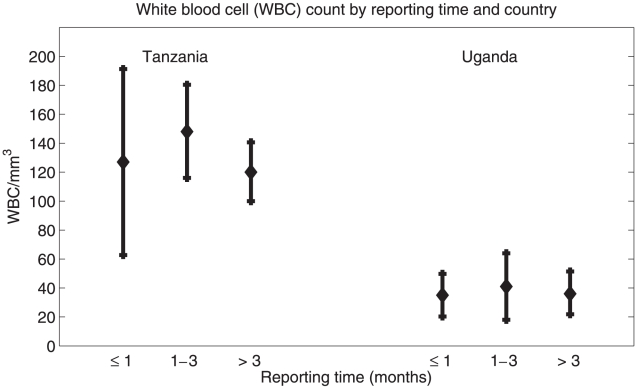
Mean and 95% confidence interval for white blood cell (WBC) count in the central nervous system (CNS) by country and reporting time.

To look at changes of diagnostic markers and clinical signs and symptoms over time we compared them in patients grouped by self-reported duration of illness (see figure 1). In Tanzania and Uganda 21.7% (15/69) and 36.2% (25/69) respectively were diagnosed with HAT having signs and symptoms for one month or less. 47.8% (33/69) of patients from Tanzania and 31.9% (22/69) of patients from Uganda were diagnosed having signs and symptoms of the disease between 1 and 3 months. Respective percentages for diagnosis of HAT after feeling ill for more than 3 months were 30.4% (21/69) in Tanzania and 31.9% (22/69) in Uganda. In both countries, the presence of trypanosomes in blood and/or CSF and the WBC count in the CSF did not significantly change over time. Also, there was no change over time for most of the clinical signs and symptoms. However, we observed that tremor (p = 0.01), walking difficulties (p = 0.040), sleeping disorders at night (p = 0.029), disturbed appetite (p = 0.044) and aggressiveness (p<0.001) aggravated over time in all patients.

Per protocol, standard assessment of co-infections at baseline included malaria and filariasis. 79.7% (55/69) of the patients from Tanzania and 2.9% (2/69) from Uganda were malaria positive on admission. None were found positive for filariasis. The HIV status was determined on voluntary basis. In Tanzania, 94.2% (65/69) of the patients tested their status and 24.6% (16/65) were found positive. In Uganda, 31.9% (22/69) tested their status and 9.1% (2/22) were found positive. We used the data from Tanzania to study implications of malaria and HIV co-infections on the clinical presentation and treatment outcomes of *T.b. rhodesiense* HAT. No significant difference either in the clinical appearance or in treatment outcomes for those patients was found. Details are shown in table 4 and 5.

**Table 4:** Clinical signs and symptoms and treatment outcomes in malaria co-infected patients from Tanzania.

	Total (n = 69)		Malaria negative (n = 14)		Malaria positive (n = 55)		p-value
	n	%	n	%	n	%	
*Clinical signs & symptoms*							
Lymphadenopathy	7	10.1	2	14.3	5	9.1	0.569
General Body Pain	69	100.0	14	100.0	55	100.0	N.A.
Headache	65	94.2	14	100.0	51	92.7	N.A.
Fever (≥37.5°C)	20	29.0	3	21.4	17	30.9	0.488
Joint pains	67	97.1	14	100.0	53	96.4	N.A.
Diarrhea	1	1.5	0	0.0	1	1.8	N.A.
Pruritus	4	5.8	3	21.4	1	1.8	0.025
Oedema	26	37.7	7	50.0	19	34.6	0.291
Dyspnoe	1	1.5	0	0.0	1	1.8	N.A.
Cough	8	11.6	2	14.3	6	10.91	0.725
Tremor	43	62.3	12	85.7	31	56.4	0.058
Hepatomegaly	4	5.8	0	0.0	4	7.3	N.A.
Splenomegaly	11	15.9	3	21.4	8	14.6	0.533
Walking difficulties	35	50.7	8	57.1	27	49.1	0.591
Abnormal movements	31	44.9	6	42.9	25	45.5	0.862
Sleeping disorder daytime	66	95.7	11	78.6	55	100.0	0.046
Sleeping disorder night time	64	94.1	11	78.6	53	96.7	0.026
Strange behaviour	15	21.7	0	0.0	15	27.3	0.001
Disturbed appetite	60	87.0	9	64.3	51	92.7	0.010
Inactivity	57	82.6	12	85.7	45	81.8	0.732
Speech impairment	6	8.7	1	7.1	5	9.1	0.818
Aggressiveness	43	62.3	7	50.0	36	65.5	0.291
*Treatment outcomes*							
Death	7	10.1	2	14.3	5	9.1	0.569
Cure (parasitological & clinical)	62	90.0	12	85.7	50	90.9	0.569

N.A: Malaria predicts presence/absence of sign and symptom perfectly.

**Table 5:** Clinical signs and symptoms and treatment outcomes of HIV co-infected patients from Tanzania.

	Total (n = 65)^a^		HIV negative (n = 49)		HIV positive (n = 16)		p-value
	n	%	n	%	n	%	
*Clinical signs & symptoms*							
Lymphadenopathy	7	10.8	5	10.2	2	12.5	0.797
General body pain	65	100.0	49	100.0	16	100.0	N.A.
Headache	61	93.9	47	95.9	14	87.5	0.247
Fever (≥37.5°C)	19	29.2	17	34.7	2	12.5	0.106
Joint pains	64	98.5	49	100.0	15	93.8	N.A.
Diarrhea	1	1.5	1	2.0	0	0	N.A.
Pruritus	2	3.1	2	4.1	0	0	N.A.
Oedema	23	35.6	17	34.7	6	37.5	0.839
Dyspnoe	65	100.0	49	100.0	16	100.0	N.A.
Cough	7	10.8	4	8.2	3	18.6	0.248
Tremor	40	61.5	31	63.3	9	56.3	0.617
Hepatomegaly	4	6.2	3	6.1	1	6.3	0.985
Splenomegaly	10	15.4	8	16.3	2	12.5	0.713
Walking difficulties	32	49.2	24	49.0	8	50.0	0.943
Abnormal movements	29	44.6	22	44.9	7	43.8	0.936
Sleeping disorder daytime	63	96.9	47	95.9	16	100.0	N.A.
Sleeping disorder night time	62	95.4	48	98.0	14	87.5	0.127
Strange behaviour	14	21.5	9	18.4	5	31.3	0.282
Disturbed appetite	57	87.7	44	89.8	13	81.3	0.373
Inactivity	53	81.5	40	81.6	13	81.3	0.973
Speech impairment	6	9.2	5	10.2	1	6.3	0.639
Aggressiveness	43	66.1	34	69.4	9	56.3	0.338
*Treatment outcomes*							
Death	7	10.8	6	12.2	1	6.3	0.510
Cure (clinical & parasitological)	58	84.1	43	87.8	15	93.8	0.510

N.A: HIV predicts presence/absence of sign and symptom perfectly,

a four (4) patients from Tanzania did not test their HIV status.

## Discussion

Based on data from the Impamel III trials we describe the clinical presentation of second stage *T.b. rhodesiense* HAT in Tanzania and Uganda and confirm a wide spectrum of clinical presentation in these two geographically distinct areas in East Africa. In both settings *T.b. rhodesiense* HAT followed the classical disease pattern, but interestingly the neurological signs and symptoms typical for HAT were seen in a relatively small percentage of patients from Uganda. In patients from Tanzania, however, they were the dominate clinical manifestation. This correlated with the significantly higher reported CSF WBC counts in patients from Tanzania.

Unspecific signs of the disease such as fever, headache, general body pain and joint pains were reported in similar proportions in both study populations. We observed fever (≥37.5) in 29.7% (41/138) of the trial subjects. In the literature, fever was reported in the range of 31–71% in second stage patients from Zambia [18], [29], [35]. In the two study populations we saw high fever (>38.5) on admission only in Uganda (5.8%, 4/69) whereof 50% were children. Fever seems to be more common in *T.b. rhodesiense* than *T.b. gambiense* second stage patients in which fever was only occasionally reported (16%) and high fever was mostly seen in children [25]. In the two study populations, oedema was reported in Uganda and Tanzania in 20.3% and in 37.7% of the patients, respectively (p = 0.0244). This was comparable to the reported range of oedema in the literature (21.7–43.3%) [18], [29], [35], [36].

The clinical aspects of *T.b. gambiense* HAT [21], [25], [26], [37] have been systematically studied and show that the hallmark of second stage disease are neurological signs and symptoms [21], [25]. Unfortunately, this has never been done for *T.b. rhodesiense* HAT and hampers comparisons. However, published data report sleeping disorders during daytime hours with 63.3–70.5% of patients being affected [29], [35]. We observed sleeping disorders during daytime hours in Uganda and Tanzania in 56.5% and in 95.7% of the patients, respectively (p<0.0001). Similarly, sleeping disorders at night time are reported in the literature in 28.3% of patients [29]. We observed it in 34.8% of the patients from Uganda and in 92.8% of the patients from Tanzania (p<0.0001). Also other neurological signs and symptoms were significantly more frequent in patients from Tanzania; tremor (p = 0.0001), abnormal movements (p<0.0001), inactivity (p = 0.0076) and aggressiveness (p<0.0001). Clearly, the neurological signs and symptoms are more pronounced in Tanzania than in Uganda, and when compared to the literature.

In Uganda, almost 50% of patients were in a poor nutritional status (48% had BMI<16.5) as food security is very poor in this part of the country. This most likely contributes to weakness and, therefore, walking difficulties in the absence of neurological symptoms. Malnutrition is associated with immunodeficiency and higher susceptibility for a wide range of infections such as tuberculosis [38], [39] and pneumonia [40], as well as a poorer response to treatment. Another potential consequence of malnutrition in Uganda is an increased number of patients admitted with severe coma indicating a more rapid progression of the disease. Yet, we assume that many HAT cases from *T.b. rhodesiense* endemic areas in Tanzania die without ever having had contact with the health system due to geographical isolation.

With regards to treatment outcomes, we did not see any differences in the two study populations. In both countries all patients were free of parasites at end of treatment. Also, there was no apparent difference in parasite clearance rates. Time- and treatment-dependant dynamics of CSF WBC counts in the two study populations will be published separately.

Cardiovascular involvement is typical, but rarely of clinical relevance in *T.b. gambiense* HAT [41], [42]. We have limited knowledge of the effects of cardiac involvement in *T.b. rhodesiense* patients, but there is evidence that perimyocarditis seems to play an important role in the clinical course and fatal outcomes [43], [44]. We observed symptoms of cardiac failure such as oedema (swelling of legs) in 29% of the patients. Hepatomegaly occurred in 18%, dyspnoea in 7% and cough in 20% of the patients. However, echocardiography or laboratory testing (i.e. brain natrium peptide) could not be performed to confirm heart failure.

Co-infections with malaria and HIV were studied in detail in the patient population from Tanzania as the majority of the patients were malaria-positive on admission (80%) and agreed to voluntary testing of their HIV status (94.2%). Patients that were malaria-positive on admission more often had pruritus (p = 0.025), sleeping disorders during day time hours (p = 0.026) and disturbed appetite (p = 0.01). Also, they exhibited strange behaviour more often (p = 0.001). However, there is insufficient evidence for profound differences in malaria-positive and malaria-negative subjects, possibly due to asymptomatic carriers.

We identified one study that looked at *T.b. rhodesiense* and HIV co-infections in 25 patients from Kenya. In terms of treatment outcomes no conclusive results were obtained [45]. Our results indicate that the HIV status of the patient does not change the clinical presentation and/or the treatment outcomes of *T.b. rhodesiense* HAT. For *T.b. gambiense* HAT, there seems to be no association between HIV and HAT infection rates [46], [47] but evidence exists for a negative association with treatment outcomes [47], [48]. More research efforts are needed to better understand the complex interactions of co- infections, especially for neglected tropical diseases [49].

Our findings on the different clinical presentation of *T.b. rhodesiense* HAT in the two study populations could be due to an observation bias, bias in patient selection, or in comparing patients at incongruous time points after infection. Bias due to co-infections or differences in host and/or parasite genetics is also possible.

An observation bias can not be ruled out but is however less likely as the Impamel III program was conducted with a structured case report form (CRF) and one monitoring person. We have seen variability in signs and symptoms with clear definitions (e.g. lymphadenopathy, abnormal movements or tremor) as well as subjective definitions (e.g. insomnia, headache or inactivity). We can not completely rule out a selection bias due to the exclusion of moribund and unconscious patients in which baseline examination per protocol was not possible. However, the number of excluded patients was relatively small (<10%) and the two study populations were similar in regards to self-reported duration of illness. Even though unsuccessful, active case searches were conducted in both countries which reduced a potential selection bias. Central nervous system involvement in *T.b. rhodesiense* HAT was previously reported within 3 weeks to 2 months of infection [12]. One third of the study population already had clear neurological signs and symptoms within one month of infection which reflects the acuteness of *T.b. rhodesiense* infections. The WBC count in the CSF as well as most of the clinical signs and symptoms also developed quickly and did not significantly change over time. Disease progression was noticeable by aggravation of tremor, walking difficulties, sleeping disorders at night time, disturbance of appetite and aggressiveness over time, in both study populations.

Based on the results shown we rule out a bias of our findings due to co-infections. Previous infections with trypanosomes and/or host genetics might be determinants for the different clinical presentation of the disease in Tanzania and Uganda. There are speculations that apathogenic forms of the disease could influence immune responses to pathogenic infections [50], [51] supported by the fact that HAT is more acute in white than in the black populations [52], [53]. But we also see a high variability in disease severity among African populations [17], [18], a fact that has been related to the descent of people: people of Nilotic descent, who migrated into the East African region from tsetse-free areas during the past 2,000 years may have less tolerance than people of Bantu descent, whose ancestors have been exposed to human trypanosomes for several thousand years [18]. Our findings do not align with this theory as in Tanzania, the majority of the population is of Bantu origin and in Uganda the majority of the population is of Nilotic origin. Different parasite genotypes could be responsible for the observed spectrum of disease severity, a hypothesis has already been raised 60 years ago [16], [17], [54]. Recent findings on the phylogenetic relationship between different *T.b. rhodesiense* strains showed that the high variability of the *T.b. rhodesiense* genome is attributed to multiple and independent evolutions from *T.b. brucei* [55]. Our data show a clear difference in the clinical presentation of *T.b. rhodesiense* HAT in Tanzania and Uganda but a detailed assessment of host and parasite genotypes was beyond the scope of this paper.

*T.b. rhodesiense* HAT is a highly neglected disease and tools for disease control are very limited. There are no sensitive diagnostics at hand and melarsoprol, the only available drug to treat second stage disease, is toxic. An early identification of the disease is vital to prevent late onset of treatment. However, most of the patients are first treated for other conditions such as malaria and pneumonia. A low degree of disease awareness among health personnel is common and aggravated by the low prevalence and the focal distribution of HAT. A detailed understanding of the clinical presentation and regional characteristics of *T.b. rhodesiense* HAT is important and can support decision making and differential diagnosis at health facility level.
